# Genome survey of nutmeg (*Myristica fragrans*) from Indonesia: genomic resource for Myristicaceae

**DOI:** 10.3389/fpls.2026.1753954

**Published:** 2026-02-04

**Authors:** Imam Civi Cartealy, Dyah Retno Wulandari, Teuku Tajuddin, Andi Salamah, Sahid Bismantoko, Ihsan Nugraha, Stevry Yushady Ch Bissa, Abinawanto Abinawanto

**Affiliations:** 1 Research Center for Computation, National Research and Innovation Agency (BRIN), Bogor, Indonesia; 2 Cellular and Molecular Mechanisms in Biological Systems (CEMBIOS) Research Group, Department of Biology, Faculty of Mathematics and Natural Sciences, Universitas Indonesia, Depok, Indonesia; 3 Research Center for Applied Botany, National Research and Innovation Agency (BRIN), Bogor, Indonesia

**Keywords:** *Myristica fragrans*, genome assembly, genome annotation, chloroplast genome, low-coverage WGS

## 1 Introduction

Nutmeg (*Myristica fragrans*), which belongs to Myristicaceae family, is a native plant of the Banda islands in Indonesia. Economically, this tree is a source for two distinct commercial spices: nutmeg and mace, which are derived from its seed and seed arils respectively. Nutmeg derived products, such as nutmeg seed extracts and essential oil, are also widely used in the food industry and in the development of pharmaceutical and medicinal products (Al-Rawi et al., 2024). For example, in the food industry, nutmeg has been used for its fragrance, although it has the potential to serve as a natural preservative to prevent foodborne pathogens (Shafi et al., 2025). In addition, nutmeg seed extracts and essential oil have been extensively studied for their medicinal properties, encompassing antibacterial, antifungal, anti-inflammatory, anticancer, antidiabetic, and antioxidant activities (Ashokkumar et al., 2022; Cruz et al., 2024; Valente et al., 2014; Jin et al., 2005; Rengasamy et al., 2018; Al-Rawi et al., 2023; Zhao et al., 2017; Nguyen et al., 2010; Adiani et al., 2015).

Despite the economic status of *M. fragrans* as commercial spices and its potential use in other areas such as medicine, genomic information of *M. fragrans* available on public repositories such as NCBI is very limited. The limitation of genetic information on *M. fragrans* hinders the research development and its sustainable conservation. Thus, information on the genomic resource of *M. fragrans* can be beneficial for further studies of *M. fragrans* that rely on genomic or genetic information. In order to unravel and drafting the genomic landscape of *M. fragrans*, we performed a low-coverage genomic sequencing of *M. fragrans* using Illumina sequencing. The genomic data had been assembled and annotated, and it provides the complete chloroplast genome and partial genome sequences of *M. fragrans*. Although the genome was partially sequenced, the data had been deposited in public repositories, adding more genomic and genetic information of *M. fragrans* and allowing public access for the benefit of future studies of *M. fragrans*.

## 2 Materials and methods

### 2.1 Plant material, DNA extraction, and sequencing

Young leaves of Myristica fragrans were collected in Bogor, Indonesia (6°38‘29.64”S,106°46’29.10”E). Genomic DNA was extracted from young leaves using the CTAB protocol (Doyle and Doyle, 1990) with modifications. The sequencing library was prepared using an Illumina DNA Prep Kit according to the manufacturer’s protocol. The quantity and quality of the library were assessed using a Qubit™ 1X dsDNA High Sensitivity Assay Kit. Finally, the library sample was loaded onto a NovaSeq 6000 (Illumina, San Diego, California) at the Genomic Facility of the National Research and Innovation Agency (BRIN), Indonesia, using a NovaSeq 6000 SP v1.5 kit (300 cycles) to generate 2x150 bp paired-end reads.

### 2.2 Genome assembly and annotation

The raw reads were processed by Fastp v0.23.2 (Chen, 2023) to remove low-quality sequences and adaptors, and to correct bases in overlapping pair-end reads. The k-mer distribution analysis was performed to estimate the genome size and heterozygosity. The frequency of k-mers was calculated using KMC v 3.2.4 (Kokot et al., 2017) with a k-mer size of 21. Then, the genome size and heterozygosity rate were estimated using GenomeScope v2.0 (Vurture et al., 2017).

The *de novo* genome assembly was performed using multiple genome assembly tools. SPAdes v4.0.0 (Bankevich et al., 2012) and Ray v2.3.1 (Boisvert et al., 2010) were used to assemble the cleaned reads with their default parameters. To produce more contiguous and complete contigs, the assembled contigs from two assemblies were merged using Redundans v2.0.1 (Pryszcz and Gabaldón, 2016). BUSCO (Manni et al., 2021) and Quast (Gurevich et al., 2013) were used to assess the quality of assembly.

Contaminant sequences were removed from the merged contigs using BlobToolKit v4.3.11 (Kumar et al., 2013; Challis et al., 2020). BlobToolKit requires the consensus, coverage, and taxonomy information of the assembly. Thus, as input to BlobToolKit, we provided the contigs, the alignment of reads to the contigs generated by BWA-MEM v0.7.17 (Li and Durbin, 2010), and taxonomy information of the contigs by searching the contigs against the NCBI non-redundant (nt) database using MMseqs2 v17-b804f (Steinegger and Söding, 2017), respectively. We identified alien contigs as any contigs that do not belong to the Streptophyta phylum nor those that do not match to any phyla.

To identify repeats, a custom repeat library for *M. fragrans* was created by combining the *de novo* repeat library generated with RepeatModeler v2.0.7 (Flynn et al., 2020) and the Viridiplantae repeat library from Dfam database v3.9 (Storer et al., 2021). RepeatMasker v4.1.9 (Tarailo-Graovac and Chen, 2009) was then used to identify and mask the repeat sequences in the assembled contigs. Augustus was run with a pre-trained gene model of *A. thaliana* to predict genes in the contigs using the masked contigs as input. To annotate the genes, MMSeqs v18.8cc5c (Steinegger and Söding, 2017) was used to match the predicted genes with the curated plant protein database from Uniprot. GO term annotations were performed by matching Uniprot IDs with their GO annotations using Uniprot ID mapping.

### 2.3 Chloroplast genome assembly and annotation

GetOrganelle v1.7.7.1 (Jin et al., 2020) was used to assemble the chloroplast genome with default parameters. Annotations of the chloroplast genome were performed using GeSeq (Tillich et al., 2017). The chloroplast genome was visualized using OGDRAW v1.3.1 (Greiner et al., 2019).

## 3 Results

### 3.1 Genome sequence and annotation

**Figure 1:**
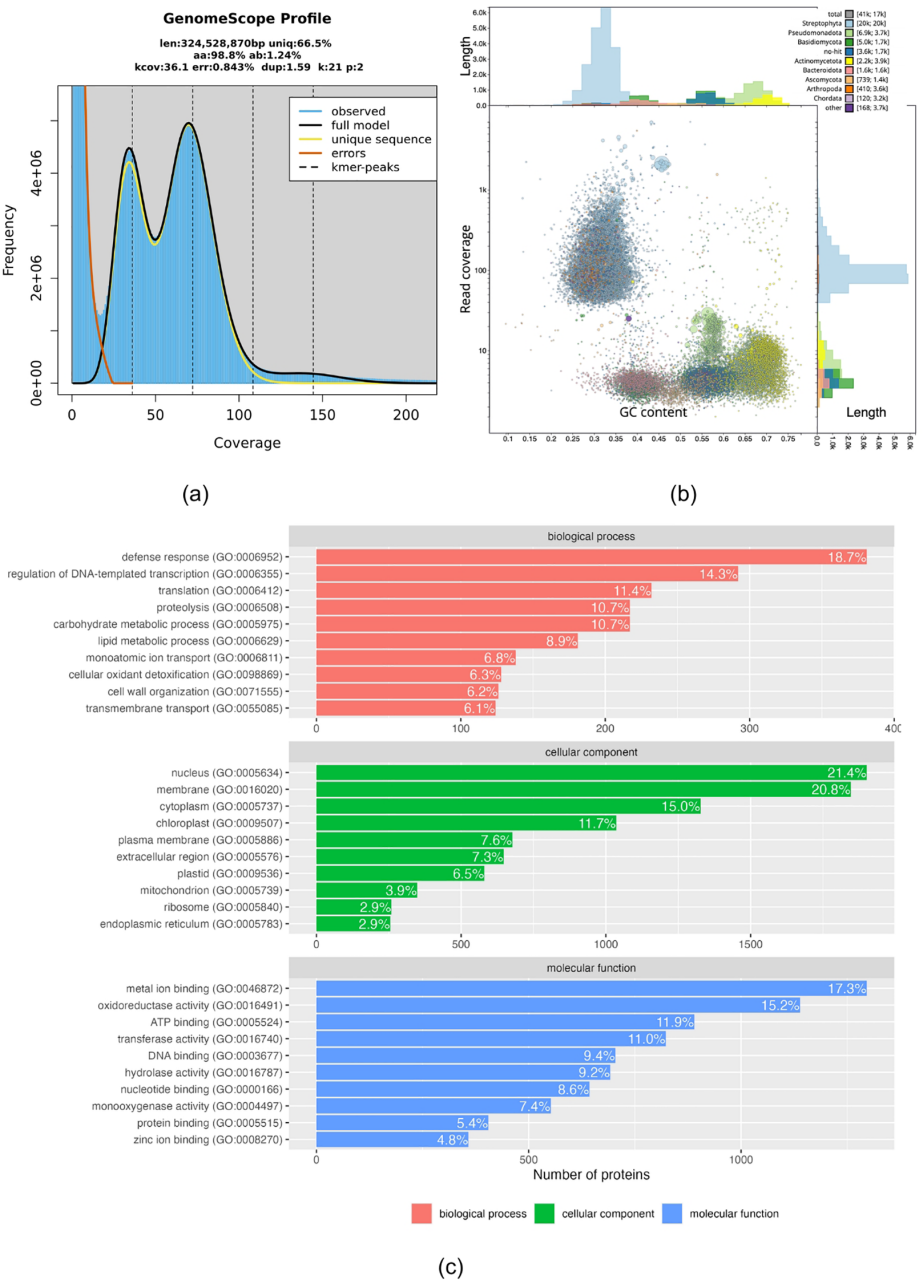
**(a)** The linier plot of k-mer coverage for 21-mer. The left peak around 40x coverage and right peak around 75x coverage are the heterozygosity and homozygosity peak respectively. **(b)** Blobplot of merged assembly from 41,225 contigs are shown. Light and dark blue contigs match to Streptophyta and ‘no hit’ respectively. Other colors represent contigs from various phyla. The top three alien contigs are shown in light green, dark green, and yellow blobs, which represent contigs from Pseudomonadota, Basidiomycota, and Actinomycetota respectively. **(c)** Bar plot of GO terms annotation of predicted proteins in *M. fragrans*. The GO terms are categorized into three ontologies, i.e. biological process, cellular component, and molecular function.

The DNA sample of *M. fragrans* generated 250.59 million paired-end reads with total bases of 36.8 Gbp. After the filtration process, a total of 227.23 million of clean paired-end reads were yielded from the raw reads (**Supplementary Table 1**). K-mer analysis estimated a genome length of 324 Mbp for *M. fragrans* (**Figure 1a**), which is lower than the flow cytometry-based estimation of *M. fragrans* genome size, i.e., between 691 and 701 Mbp/1C (Kuo et al., 2024). This indicates that the genome of *M. fragrans* was partially sequenced, which is likely due to the low sequencing coverage. We estimated the average read coverage is 52x using the maximum estimated genome size of 701Mbp. The reads fit the GenomeScope v2 model for diploid species with repeat sequences and heterozygosity percentage of 33.5% and 1.24% respectively (**Figure 1a**).

To assemble the *M. fragrans* genome, we used an ensemble method which combines multiple assemblers to generate more contiguous assembly. Individually, Ray generated an assembly of 288 Mbp in 47,091 contigs, while SPAdes generated 324 Mbp in 75,430 contigs. We merged the outputs of Ray and SPAdes, and removed heterozygous contigs using Redundans to produce homozygous scaffolds. These steps generated a more homozygous and contiguous merged assembly of 301 Mbp in 41,225 contigs. However, we detected alien contigs in the 301 Mbp of merged assembly (**Figure 1b**). Alien contigs contribute to almost half (41.85%) of the total number of contigs, but they only account for 40 Mbp (13.51%) of the assembly length. The top three alien contigs in the merged assembly ranked by the number of contigs are Pseudomonadota (16.80%), Basidiomycota (12.22%), and Actinomycetota (5.4%). After removing the alien contigs, the final assembly yielded a total of 260 Mbp in 23,973 contigs (**Supplementary Table 2**).

Augustus detected 145,534 exons in the masked assembly, which formed 30,340 proteins. To determine the identity of these proteins, we compare their sequences to the curated Uniprot plant protein sequences. Of the predicted proteins, 20,453 identity matches were found, representing 67.4% of the total predicted proteins. **Figure 1c** shows the distribution of GO terms of the identified proteins in the *M. fragrans* assembly.

### 3.2 Chloroplast genome sequence and annotation

**Figure 2:**
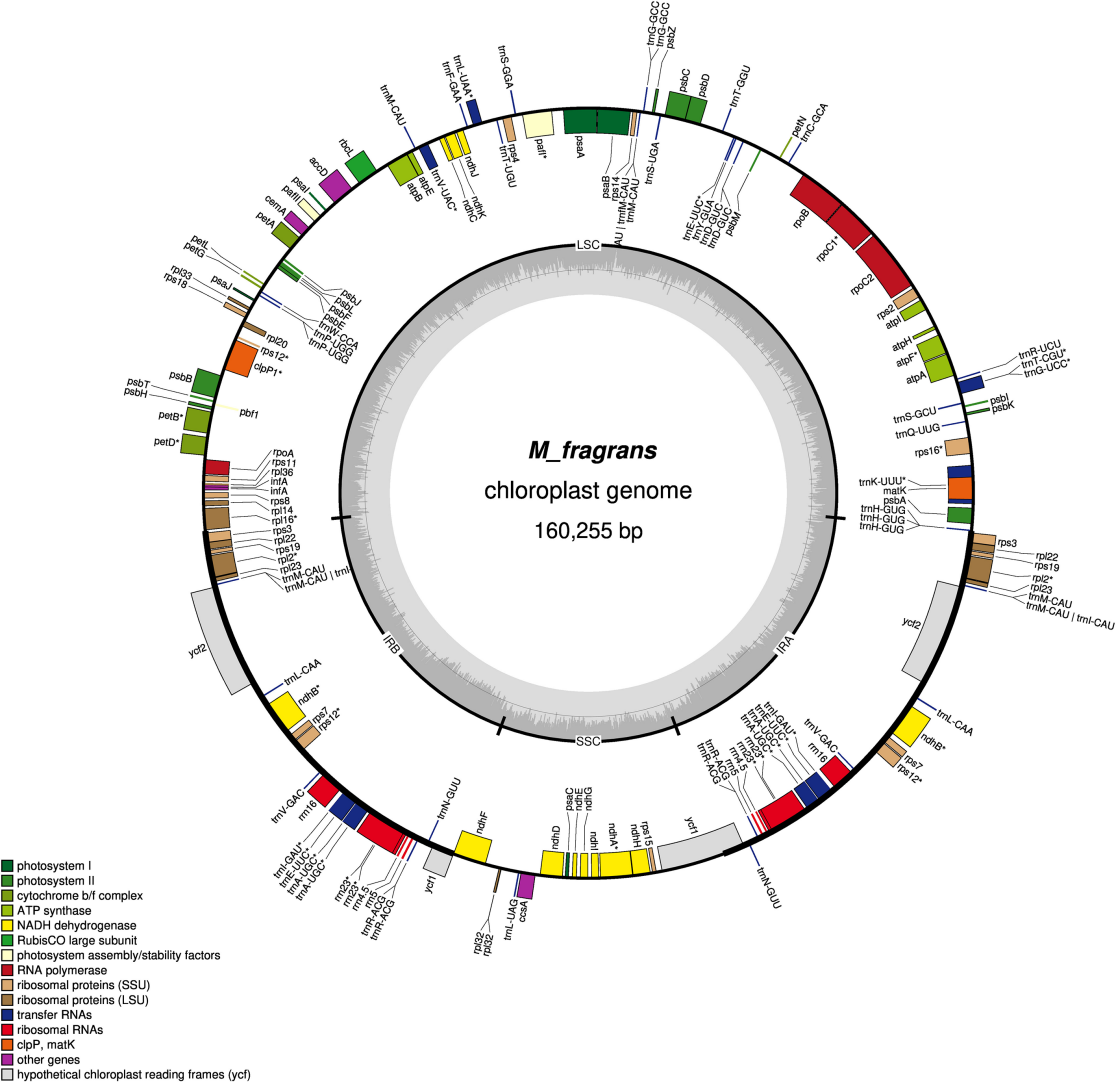
The chloroplast genome of *M. fragrans*.

The size of the chloroplast genome of *M. fragrans* is 160,255 bp (**Figure 2**). It has a typical quadripartite chloroplast structure consisting of a pair of inverted repeats (IRs) of 28,403 bp separated two single copy regions, the large single copy (LSC) of 85,160 bp and small single copy (SSC) of 18,289 bp. The chloroplast genome contains 134 genes, which are categorized based on their function in the plastid as described in **Supplementary Table 3**. It consists of 8 ribosomal RNA genes, 37 transfer RNA genes, and 89 protein coding genes (PCG). Of these, 21 genes are duplicated in the IR regions.

In comparison to previously published chloroplast genome of *M. fragrans* (Cai et al., 2019), which reported the chloroplast genome size of 155kbp, the size of the chloroplast genome of *M. fragrans* reported in this publication is longer (**Supplementary Table 4**). We also identified two additional chloroplast genes, which are present in the chloroplast genome reported in this publication, i.e. the t-RNA gene *trnG-GCC* and the large subunit of ribosome gene *rpl22*, but are missing in chloroplast genome reported by (Cai et al., 2019).

## Data Availability

The datasets presented in this study can be found in online repositories. The names of the repository/repositories and accession number(s) can be found below: https://www.ncbi.nlm.nih.gov/SRX30845511https://www.ncbi.nlm.nih.gov/genbank/PX562784https://figshare.com/ 10.6084/m9.figshare.30585914.
