# Supplementary material for: Genome survey of nutmeg (Myristica fragrans) from Indonesia: genomic resource for Myristicaceae
Source: Front Plant Sci. 2026 Feb 4;17:1753954. doi: 10.3389/fpls.2026.1753954 (PMC12913573; doi:10.3389/fpls.2026.1753954)
Supplement: Supplementary file 1 [file Table1.docx]

Table 1. *M. fragrans* reads summary

|  | Reads summary |
| --- | --- |
| Bioproject | PRJNA1345510 |
| Biosample | SAMN52751313 |
| Accession Number | SRX30845511 |
| Total reads | 250,596,296 |
| Total bases | 36,804,866,603 |
| q20 bases | 33,518,007,122 |
| q30 bases | 29,931,660,074 |
| q20 rate | 0.910695 |
| q30 rate | 0.813253 |
| Read1 mean length | 147 |
| Read2 mean length | 146 |
| GC content | 0.358959 |

Table 2. Assembly statistics of various assemblers

|  | Ray | Spades | Merged | Final assembly |
| --- | --- | --- | --- | --- |
| Total length (bp) | 288,192,000 | 324,186,996 | 301,638,457 | 260,889,073 |
| Largest contig (bp) | 186,763 | 379,939 | 379,939 | 379,939 |
| Contigs | 47,091 | 75,430 | 41,225 | 23,973 |
| N50 AVG_len (bp) | 10,955 | 8,151 | 16,914 | 19,414 |
| BUSCO | 88.6% | 75.0% | 88.7% | 91.4% |

Table 3. Gene composition of the complete chloroplast genomes of *M. fragrans*.

| Name | Value | Total |
| --- | --- | --- |
| Small subunit of ribosome | rps11, rps12 (x2), rps14, rps15, rps16, rps18, rps19 (x2), rps2, rps3 (x2), rps4, rps7 (x2), rps8 | 16 |
| tRNA | trnA-UGC (x2), trnC-GCA, trnD-GUC, trnE-UUC, trnF-GAA, trnG-GCC, trnG-UCC, trnH-GUG, trnI-CAU (x2), trnI-GAU (x2), trnK-UUU, trnL-CAA (x2), trnL-UAA, trnL-UAG, trnM-CAU, trnN-GUU (x2), trnP-UGG, trnQ-UUG, trnR-ACG (x2), trnR-UCU, trnS-GCU, trnS-GGA, trnS-UGA, trnT-GGU, trnT-UGU, trnV-GAC (x2), trnV-UAC, trnW-CCA, trnY-GUA, trnfM-CAU | 37 |
| Photosystem II | psbA, psbB, psbC, psbD, psbE, psbF, psbH, psbI, psbJ, psbK, psbL, psbM, psbT, psbZ | 14 |
| Maturase | matK | 1 |
| Subunit of synthase | atpA, atpB, atpE, atpF, atpH, atpI | 6 |
| DNA dependent RNA polymerase | rpoA, rpoB, rpoC1, rpoC2 | 4 |
| Subunit of cytochrome | petA, petB, petD, petG, petL, petN | 6 |
| Photosystem I | psaA, psaB, psaC, psaI, psaJ | 5 |
| Photosystem I and II assembly | pafI, pafII | 2 |
| NADH dehydrogenase | ndhA, ndhB (x2), ndhC, ndhD, ndhE, ndhF, ndhG, ndhH, ndhI, ndhJ, ndhK | 12 |
| Large subunit of rubisco | rbcL | 1 |
| Subunit acetyl-coA carboxylase | accD | 1 |
| cp envelope membrane protein | cemA | 1 |
| Large subunit of ribosome | rpl14, rpl16, rpl2 (x2), rpl20, rpl22 (x2), rpl23 (x2), rpl32, rpl33, rpl36 | 12 |
| ATP-dependent protease subunit P | clpP1 | 1 |
| Photosystembiogenesis factor 1 | pbf1 | 1 |
| Translation initiation factor | infA | 1 |
| Component of 2-MD heteromeric AAA-ATPase complex | ycf2 (x2) | 2 |
| rRNA | rrn16 (x2), rrn23 (x2), rrn4.5 (x2), rrn5 (x2) | 8 |
| Component of TIC complex | ycf1 (x2) | 2 |
| C-type cytochrome synthesis | ccsA | 1 |

Table 4. Comparison of the published chloroplast genome features of *M. fragrans*.

|  | *M. fragrans* by (Cai et al. 2019) | *M. fragrans* (this publication |
| --- | --- | --- |
| Accession number | MN495963 | PX562784 |
| Genom size (bp) | 155,868 | 160,255 |
| GC contents (%) | 39.2 | 37.9 |
| LSC length (bp) | 87,062 | 85,160 |
| SSC length (bp) | 20,664 | 18,289 |
| IR length (bp) | 24,071 | 28,403 |
| Number of genes | 126 | 134 |
| Number of genes (no duplicate) | 111 | 113 |
| Number of rRNAs | 8 | 8 |
| Number of tRNAs | 35 | 37 |

References

Cai, Chao‐Nan, Hui Ma, Xiuqin Ci, John Conran, and Jie Li. 2019. “Comparative Phylogenetic Analyses of Chinese Horsfieldia (Myristicaceae) Using Complete Chloroplast Genome Sequences.” *Journal of Systematics and Evolution* 59 (December). https://doi.org/10.1111/jse.12556.
